# Production and Characterization of Heme Iron Polypeptide from the Blood of Skipjack Tuna (*Katsuwonus pelamis*) Using Enzymatic Hydrolysis for Food Supplement Application

**DOI:** 10.3390/foods12173249

**Published:** 2023-08-29

**Authors:** Satita Tansukkasem, Piriya Kaewpathomsri, Veasarach Jonjaroen, Panwajee Payongsri, Sittiwat Lertsiri, Nuttawee Niamsiri

**Affiliations:** 1 School of Bioinnovation and Bio-Based Product Intelligence, Faculty of Science, Mahidol University, Rama 6 Road, Bangkok 10400, Thailand; 2 Global Innovation Center (GIC), Thai Union Group PCL., S.M. Tower, Phaholyothin Road, Phayathai Sub-District, Phayathai, Bangkok 10400, Thailand; 3 Department of Biotechnology, Faculty of Science, Mahidol University, Rama 6 Road, Bangkok 10400, Thailand

**Keywords:** skipjack tuna blood, heme iron polypeptide (HIP), enzymatic hydrolysis, degree of hydrolysis, solubility, iron supplement

## Abstract

Organic heme iron in the form of heme iron polypeptide (HIP) is a bioavailable form of iron that can be used for dietary supplements. However, one practical challenge with HIP is that the quality of HIP prepared with different batches of raw material could lead to HIP products with inconsistent characteristics. In this study, skipjack tuna blood, a by-product in canned tuna industry, was converted to HIP at different degrees of enzymatic hydrolysis. The variation in HIP physical–chemical characteristics from different batches was evaluated, including composition, solubility, and molecular weight distribution. It was found that the batch variation had no effect on HIP composition and solubility; however, the degree of hydrolysis (DH) and the size of peptides that interact with heme greatly influenced HIP solubility at pH 2. Tuna-HIP with a low DH (DH, 8%) had 1.76-fold greater solubility than tuna-HIP with a high DH (DH, 32%). High-performance liquid chromatography (HPLC) revealed that tuna-HIP with a low DH had a molecular weight ranging from 1 kDa to 5 kDa. In summary, HIP-derived tuna blood was found to contain 70.54 ± 3.22 mg/100 g of iron and exhibit good solubility at 58.0 ± 2.16% at pH 2. Thus, tuna-HIP with a low DH might be a suitable functional ingredient for iron fortification of food.

## 1. Introduction

Iron deficiency is one of the major global malnutrition concerns, estimated to contribute to 50% of anemia cases worldwide [1,2]. Iron is important for aerobic metabolism as it is a key component of hemoglobin and myoglobin, and also an essential co-factor in heme proteins involved in immune function [3]. A low level of iron in the blood can cause fatigue, shortness of breath, and lead to a general decline in physical health. Iron can be obtained from dietary foods in two principal forms: organic iron (i.e., heme iron) and inorganic iron (i.e., ferrous (Fe (II) and ferric (Fe (III) salts). Among these, heme iron is considered a more effective iron supplement because of its fewer gastrointestinal side effects and higher bioavailability compared to non-heme iron [1]. Common sources of organic iron include meat or blood by-products because they naturally contain high concentration of heme-containing proteins such as myoglobin and hemoglobin [4]. The heme molecule is hydrophobic and thus it is poorly water soluble in its free form. Purified heme also suffers from a high chance of heme polymerization, especially under gastric conditions at pH 2, and this subsequently leads to its low bioavailability. To enhance solubility, heme iron polypeptide (HIP) has been proposed as an alternative form for iron supplementation.

Heme iron polypeptide (HIP) is commonly produced from porcine or bovine blood using enzymatic hydrolysis. In HIP, heme iron is bound to peptides derived from the digested hemoglobin. Previous studies have illustrated that HIP exhibit good solubility at pH 2 but that the MW of the associated peptides varies depending on the sources and preparation. These include 2–3.6 kDa in fish HIP [5], 4–5 kDa in bovine HIP [6], 1–7.5 kDa in porcine HIP [7], and 3–14.4 kDa in porcine HIP [8]. Compared to pure heme, HIP could lead to 10 times higher heme iron absorption [5,6]. However, one challenge of HIP as an effective iron supplement is the consistency of HIP preparations. The key characteristics of HIP preparations depend on the degree of hydrolysis, the strength of the heme–peptide interaction, heme polymerization, heme iron content, peptide content, and solubility under gastric condition (pH 2) [9]. The properties of final HIP products can be greatly affected by the production process, including blood sources, types of hydrolytic enzymes, pH, and hydrolysis time [7,10]. Due to religious beliefs, HIP derived from porcine and bovine sources might not be accepted by some groups and individuals, and thus, there is interest in other raw material sources, including fish. The production of fish-HIP from cultured yellowtail fish hemoglobin demonstrated that fish blood could be used for HIP preparation, resulting in fish-HIP peptides with an MW of 2.0 to 3.6 kDa with a high heme iron content (5.1% dry basis) and an apparent solubility of 88% [5]. These characteristics of fish-HIP were found to be similar to HIP prepared from mammalian hemoglobin and can be considered an alternative good source of heme iron supplements.

Thailand is one of the major global exporters of canned tuna, with more than 800,000 MT of tuna processed in a year [11]. The main species of tuna that are captured and used for canned tuna processing are skipjack (*Katsuwonus pelamis*), yellowfin (*Thunnus albacares*), and bigeye (*Thunnus obesus*). Skipjack tuna represent the largest volume of species used for canning, and it is also considered the most sustainable tuna species as its population numbers are stable because it is growing fast and can reproduce from 2 years of age (International Seafood Sustainability Foundation (ISSF), 2023). Typically, tuna-canning procedures yield over 60% by-products consisting of solid wastes (25–30%), such as bones, heads, gills, dark meat, and viscera, as well as liquid wastes (30–35%), which primarily consist of blood (7–12%) and cooked tuna juice (10–14%). These by-products contain a variety of organic compounds, including hemoglobin, proteins, and lipids, which have numerous potential applications [11]. Thus, tuna blood could be considered as a valuable starting material for HIP production. Nevertheless, there are some considerations to keep in mind using tuna blood for producing high-quality HIP. First, tuna blood must be collected in a defrosted state [12]. Thus, the composition and quality of tuna blood may be different from blood derived from freshly slaughtered animals where the blood contains intact cells. Second, tuna fish are typically harvested in mass quantities. There might be variation between batches due to seasonal changes and fishing areas that could influence the quality of tuna blood by-products [13]. Additionally, since tuna must be caught from the ocean and subsequently stored under frozen conditions until processing, the frozen storage period may also affect the tuna biochemical quality [14].

This study aimed to analyze the properties of water-soluble heme iron polypeptide (HIP) derived from tuna blood using a commercially available enzyme Alcalase. Three different batches of tuna blood were collected, and the effect of batch variation on HIP product characteristics was investigated. Blood hydrolysates were also collected at different times (0.5, 1, 2, 3, and 4 h) to understand the effect of the degree of hydrolysis on tuna-HIP characteristics and solubility. In addition, high-performance liquid chromatography (HPLC) and UV–Vis spectroscopy were used for determining the molecular weight of tuna-HIP and the heme iron peptide binding, respectively.

## 2. Materials and Methods

### 2.1. Chemicals and Reagents

The Alcalase 2.4 L FG enzyme obtained from Brenntag Ingredient PCL. (Sathon, Bangkok, Thailand) was used to hydrolyze the proteins in tuna blood. Hematin was purchased from Sigma-Aldrich (St. Louis, MO, USA). All other reagents used were of analytical grade.

### 2.2. Tuna Blood Preparation

This study utilized frozen tuna obtained from tuna canned processing of Thai Union Manufacturing Co., Ltd. (Samut Sakhon, Thailand). Three different batches of tuna collected from different fishing trips were used as shown in Table 1. Skipjack tuna (*Katsuwonus pelamis*) was beheaded from the factory in a frozen condition, then kept in the ice box for delivery to laboratory and thawed at 4 °C for 24 h. Subsequently, the mid-head thawed tuna was centrifuged for 5 min per round to collect tuna blood (mainly from gills) and kept at −20 °C until use.

### 2.3. Tuna-HIP Production

Tuna-HIP was prepared by enzymatic hydrolysis according to the method adapted from the previous studies [5,6]. Briefly, tuna blood (500 mL) was stirred with an overhead stirrer (IKA RW 20 digital) at 55 °C for 45 min to pre-incubate. The pH was adjusted to pH 8.5 with NaOH in order to prepare the condition for hydrolysis. Alcalase was then added to the mixture at 1% E/S protein and the digestion was carried out for 4 h at 55 °C with constant stirring. Blood hydrolysate was collected at 0, 0.5, 1, 2, 3 and 4 h for analysis. At each time point, the mixture was heated at 90 °C for 30 min to inactivate Alcalase. Subsequently, the samples from different hydrolysis time were freeze-dried to obtain the tuna-HIP powder using a freeze dryer (Alpha 2–4 LDplus, CHRIST, Osterode am Harz, Germany) at −80 °C for 72 h.

### 2.4. Determination of Degree of Hydrolysis of HIP

The degree of hydrolysis of tuna-HIP in different hydrolysis times were determined using the trinitrobenzenesulfonic acid (TNBS) method according to a previous study [15] and calculated via an equivalent L-leucine standard. Tuna-HIP (50 µL) was gently mixed with 500 μL of a 0.2125 M phosphate buffer (pH 8.2) and a 0.05% TNBS reagent. The mixture was incubated at 50 °C for 1 h; then, the incubation was stopped by adding 1 mL of 0.1 N HCl. The mixture was incubated at room temperature in the dark for 30 min. The reaction was measured by its absorbance at 420 nm by UV–Vis spectrophotometer (UP2900, Hitashi, Tokyo, Japan). The degree of hydrolysis was calculated using equation

DH% = [(L_t_ − L_0_)/(L_max_ − L_0_)] × 100, (1)

where L_t_ is the total number of α-amino groups measured at time t. L_0_ is the total number of α-amino groups at t_0_. L_max_ is the total number of α-amino groups obtained after hydrolysis using 6 N of the HCl solution at 110 °C for 24 h.

### 2.5. Tuna Blood and Tuna-HIP Powder Composition Analysis

The compositions of tuna blood and tuna-HIP powder were examined for total iron by the atomic absorption spectrophotometer method [16] and for heme iron by the pyridine hemochromagen method [17]. Protein was determined for tuna blood by the Kjeldahl and Lowry method according to the standard method of proximate analysis (AOAC, 2007) [18].

### 2.6. Water Solubility Analysis of HIP

To evaluate the apparent water solubility of tuna-HIP and hemin, the method based on a previous fish-HIP study [5] was used with some modifications. Briefly, each sample (10 mg) was added to 15 mL of different buffer solutions (pH 2–12); citrate, phosphate, and borate buffers at a pH ranging from 2 to 12, accordingly. The aqueous mixtures were mixed using vortex and shaken at 30 °C (120 rpm) for 24 h. Then, the mixtures were centrifuged at 10,000× *g* for 5 min to separate the aqueous solution and the pellet. The supernatants were collected for heme iron determination by measuring the absorption at 395 nm with a UV–Vis spectrophotometer [19]. The hematin standard curve was used to quantify soluble heme. Relative solubility was calculated by equation

(2) $$\%\mathrm{Relative}\;\mathrm{solubility}\;=\frac{\mathrm{Heme}\;\mathrm{solubility}\;\mathrm{of}\;\mathrm{HIP}\;\mathrm{at}\;\mathrm{each}\;\mathrm{pH}\;(µg)}{\mathrm{Heme}\;\mathrm{solubility}\;\mathrm{of}\;\mathrm{HIP}\;\mathrm{at}\;\mathrm{pH}\;12(µg)}\times 100.$$

### 2.7. HIP Molecular Weight Distribution Analysis

The molecular weight distribution of heme iron polypeptide was estimated by gel permeation chromatography with UV detection at optical densities of 220 nm and 395 nm. The gel filtration column, TSKgel^®^G2000 SWXL 7.8 × 300 mm column (Tosoh, Tokyo, Japan), was used. The system was a 1260 Infinity system equipped with a diode-array detector (Agilent, Santa Clara, CA, USA) [20]. The mobile phase consisted of 45% acetronitile-0.1% TFA at a flow rate of 0.5 mL min^−1^. The column temperature was 30 °C. The correlation between the molecular weight (Log_MW_) and retention volume (R_v_) were determined by gel permeation chromatography of the following protein marker weight consisting of Thyroglobulin (MW: 669 kDa), Cytochrome C from bovine heart (MW: 12 kDa), Aprotinin from bovine lung (MW: 6511 Da), Bacitracin (MW: 1423 Da), N-Hippuryl-His-Leu hydrate powder (HHL) (MW: 429 Da), and Gly-Gly (MW: 132 Da).

### 2.8. UV–Visible Difference Spectrometry

Twenty milligrams of hematin and HIP were separately dissolved in a 0.1 M NaOH and diluted to 25 μM with a 0.01 M phosphate buffer, pH 7.5 [6]. Absorption spectra were recorded on a spectrophotometer (Jasco V-700, Easton, MD, USA) between 300 and 500 nm to determine the presence of the heme–peptide complex in HIP.

### 2.9. Statistical Analysis

All results were expressed as mean ± standard deviation (SD). Analysis of variance (one-way ANOVA) was used to assess statistical difference between protein and iron content of tuna blood. The paired sample *t*-test was used to access the statistical differences between solubility of heme iron polypeptide powder in different time treatments followed by the least significance difference test to evaluate differences between sets of mean values. The significance level was set at *p* < 0.05. All analyses of data were carried out using SPSS Statistics for Windows version 18 (SPSS Inc., Chicago, IL, USA).

## 3. Results and Discussion

### 3.1. Effect of Tuna Batch Variation on Blood Composition and HIP Characteristics

Heme iron polypeptide (HIP) could be produced from a variety of blood sources including fish and mammalian blood. Generally, once tuna is caught, it is kept frozen for more than a month. The thawed blood is then collected after the freeze–thawing process. This makes the production process quite different from mammalian and farmed-fish blood harvesting, where the blood can be collected immediately after slaughter. The pre-production process and fish variation could affect tissue quality and subsequently blood quality including heme and iron content. As a result, the effect of tuna batch variations on the composition of the collected blood was evaluated to understand whether this would have an impact on final HIP characteristics including protein, total iron, heme, and heme iron. 

#### 3.1.1. Effect of Tuna Batches on Blood Composition

The compositions of protein, total iron, heme, and heme iron in tuna blood from different batches were evaluated (Table 2). Protein content varied between 58.87 and 65.99 g/100 g (dry basis), and the differences were statistically significant among three batches (*p* < 0.05). In contrast, the total iron contents and heme iron contents were similar across all three batches, at around 70 mg/100 g (0.07%) and ranging from 52.60 to 54.76 mg/100 g, (dry basis), respectively. The percentage of heme iron was ranging from 75% to 78%, and no significant differences were observed (*p* > 0.05).

There could be many possible reasons for protein differences in the tuna blood collected from forty tuna heads in each batch. Previous studies also showed that the amount of protein and other proximate components in fish tissue and blood might depend on age and size of the fish, sexual maturity of the fish, as well as geographic location of the catch [21,22]. 

In the Yuenyongputtakal [23] study, it was revealed that tuna blood by-products have the potential to become a source of protein and iron due to protein and iron content similar to commercial sources. The authors collected blood from the canned tuna (*Katsuwonus pelamis*) production line and kept it frozen at −20 °C until processing into blood powder. Their results showed total iron content of 0.12% dry basis, which was slightly lower than the total iron content of blood from porcine, chicken, and duck at approximately 0.18% [24]. 

However, our study showed total iron content of 0.07%, which is much lower than other sources previously mentioned. It might be possible that during the freezing and thawing process, ice crystals could rupture red blood cells. Consequently, fewer intact cells are available to enclose the soluble hemoglobin and the heme–iron complex, resulting in a decrease in the amount of total iron and heme iron in the thawed blood after centrifugation. Such phenomena were illustrated in another study where the effect of multiple freeze–thaw cycles on total and heme iron contents of bonito (*Sarda sarda*) and bluefish (*Pomatomus saltator*) fillets were investigated. The authors showed that when the number of freeze–thaw cycles of fish increased, the concentration of total iron and heme iron significantly decreased, leading to reduction in the total iron content. It is also possible that the reduction in the total iron content might be due to the drip loss during the thawing process, leading to a decrease in heme iron [25]. In our study, the mid-head of tuna was thawed before blood collection, as it contains proteins and iron, which are susceptible to lipid oxidation. In another study, the authors investigated the effect of hydrogen peroxide and hemin on the changes in myoglobin from the dark muscle of Eastern little tuna (*Euthynnus affinis*). Their results suggested that hydrogen peroxide, which could oxidize, could modify the redox state of myoglobin and alter the structure of the globin protein. Consequently, this could enhance the release of iron from the porphyrin ring [26].

#### 3.1.2. Effect of Tuna Batches on Degree of Hydrolysis, Iron Contents and Solubility of HIP 

**Figure 1:**
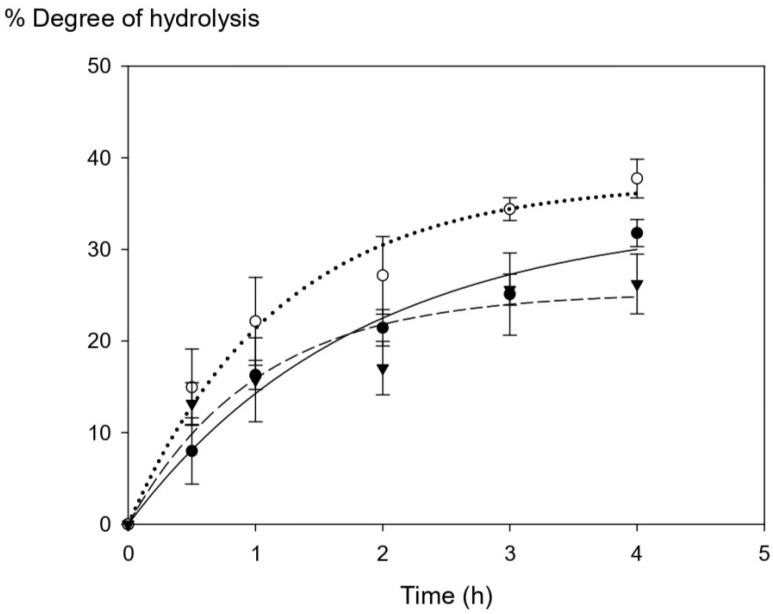
The changes in the degree of hydrolysis (DH) of tuna blood at different time points (Batch A = ⬤, Batch B = ◯, and Batch C = ▼). The graph was produced by SigmaPlot version free trial, curve fitting with exponential rise to maximum. Statistical analysis was performed using One-way ANOVA, *p* = 0.05, comparing each variation batches.

Tuna blood protein was collected from different batches and subjected to enzymatic hydrolysis using commercial Alcalase over different times (0.5 h–4 h) in order to digest the protein-associated heme iron into smaller-size peptides to improve heme solubility. Figure 1 shows the progression of the degree of hydrolysis upon enzymatic digestion. The degree of hydrolysis (DH) within the first hour was in the range of 7.99–14.96% with no significant difference among three tuna batches (*p* > 0.05). However, the DH values started to be different among three batches after 2 h of hydrolysis time. The maximum ranges of DH at the highest hydrolysis time (4 h) were within 26–38%, and they were statistically significantly different (*p* > 0.05) among Batch A, B, and C. Overall, our results show a higher DH range (8–37%) compared with that of other studies from porcine and bovine hemoglobin, which have a range of DH of 6–22% [6,7].

There are many possible explanations for the final DH differences among different batches of tuna blood which may not be concluded at this point. The degree of hydrolysis is proportional to the amount of cleaved peptide bonds in the protein hydrolysate [27]. One possible explanation might be the fact that DH could be affected by low solubility of starting HIP and their associated protein chains to be fully susceptible to enzymatic cleavage during the hydrolysis process, thus leading to final DH variations observed [6]. Protein and peptide precipitation cannot be ruled out.

To evaluate the stability of heme and heme iron after enzymatic hydrolysis, the HIP powders obtained after 4 h of hydrolysis time were analyzed for iron, heme, and heme iron content compared to the initial content in the start material as shown in Table 3. The results showed that the total iron and heme iron contents of tuna-HIP powder were ranging from 67.71 to 70.92 mg/100 g and 51.71 to 55.06 mg/100 g, respectively. The values were not significantly different from those in the initial tuna blood (*p* > 0.05). However, when compared with HIP from another fish source [5], tuna-HIP showed a lower heme iron content, consistent with the lower iron content (dry basis). In yellowtail fish blood, HIP had a heme iron content ranging from 2.1 to 5.1 g/100 g (dry basis) after being concentrated via ultrafiltration. There might be several possible explanations for the lower heme iron content of our tuna-HIP. First, the lower heme iron content in tuna-HIP might be due to the fact that preparation process of tuna-HIP does not allow for collection and concentration of intact erythrocytes. The cells are ruptured during the freeze–thaw process, leading to a release of heme iron proteins into the serum or plasma. Additionally, our study did not include the ultrafiltration process to concentrate and discard unnecessary proteins, peptides and inorganic salts, which could dilute the overall heme iron concentration. 

**Figure 2:**
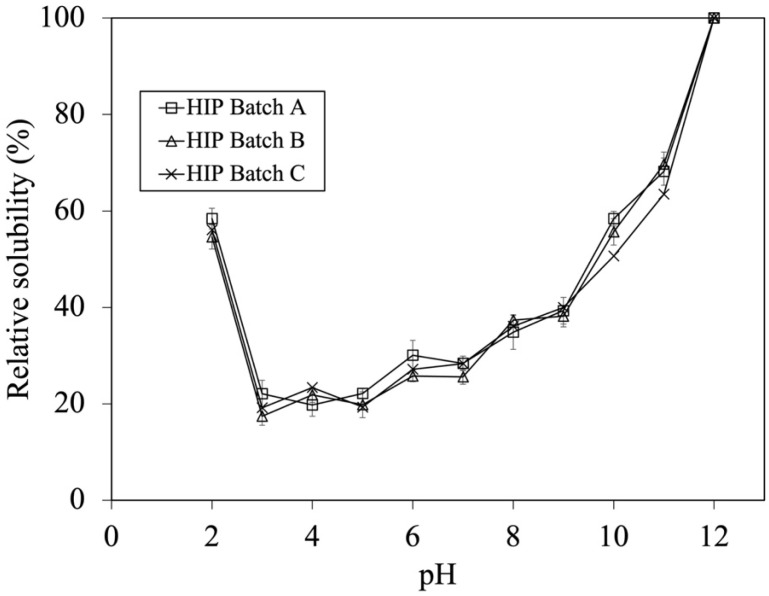
Effect of batch variation on tuna-HIP from 0.5 h solubility at pH 2–12. Statistical analysis was performed using One-way ANOVA, *p* = 0.05, comparing each variation batches.

As mentioned above, batch variation did not affect the DH during the first hour of hydrolysis. However, their solubility profile should be compared in order to confirm their similarity. From previous studies, the work of Liu et al. (2010) illustrated that HIP with enhanced solubility at pH 2 possess peptides with MW between 1 and 7.5 kDa [6,7]. As a result, the HIP powder subjected to a 0.5 h enzymatic hydrolysis (i.e., containing DH in a range of 8–15%) was initially used for studying the effect of tuna blood batch variation on HIP solubility. The solubility profiles of HIP from Batch A, B, and C at the pH ranging from 2 to 12 are shown in Figure 2. 

The calculation of percent solubility was performed by comparing the solubility of each HIP (0.5 h) batch at various pH values to its maximum solubility at pH 12. The solubility trends of HIP from different tuna batches were not significantly different (*p* > 0.05). This illustrated that batch variation did not affect the solubility of HIP when the DH was between 8 and 15%, which could be the key parameter for quality control during industrial manufacturing. The ranges of relative solubilities of HIP are 55–58%, 17–23%, 26–30%, 36–40%, 46–58%, and 63–70% at gastric conditions (pH 2), weak acidic conditions (pH, 3–5), neutral conditions (pH, 6–7.5), and basic conditions (pH, 8–9, and pH, 10–11), respectively, as shown in Figure 2. In addition, this finding suggests that tuna-HIP from different batches with a %DH in a range of 7–15% might be sufficient for making HIP soluble (up to 60%) at gastric stomach pH 2. Generally, most of iron is taken up in the first part of the small intestine, primarily by the duodenum and the upper part of the jejunum. Nonetheless, small quantities of iron could also be assimilated from the stomach, ileum, and colon [28]. The condition of pH in the small intestine ranges from 7.5 to 8. Although our study discovered that the relative solubility values of tuna-HIP at a pH from 7 to 8 ranges were at around 30–40%, this should still be sufficient for the absorption of heme iron in the small intestine. This is because the small intestine has the capability to utilize different forms of iron. The brush border membrane has various transporters that can facilitate the absorption of heme iron [29]. On the other hand, studies have showed that the resulting heme iron molecules from digested hemoglobin under stomach condition were still poorly soluble at low pH values of the stomach. This subsequently could reduce their absorption in the intestine [8,30,31]. However, our study could yield tuna-HIP with high solubility of around 60% at pH 2 that might possibly promote better iron absorption under physiological conditions. Previous studies also showed that when HIP was produced by low DH at around 7–15%, their solubility at pH 2 was significantly improved. For example, bovine-HIP with a DH of 11% and porcine-HIP with a DH of 8–12% demonstrated better solubility at pH 2 [6,7]. 

### 3.2. Effect of Degree of Hydrolysis on Solubility and Molecular Weight of Tuna-HIP

Since tuna-HIP was produced by enzymatic hydrolysis, this resulted in increasing the %DH and shortening the peptide size of tuna-HIP as the reaction time progressed. According to previous studies [7,9,10], both %DH and peptide sizes could have a great effect on HIP solubility. In one study, high %DH at around 23% (i.e., yielding smaller peptides) could adversely decrease heme solubility [10]. On the contrary, another study showed that low DH at around 8% (1–7.5 kDa) (i.e., yielding larger peptides) could improve heme solubility [7]. In order to find the optimal digestion time, the effects of low and high %DH of tuna-HIP (i.e., 8% and 32% DH for Batch A, 15% and 38% DH for Batch B and 13% and 26% DH for Batch C) on the relative heme solubility were investigated. The relative solubilities of tuna-HIP from three different batches (Batch A, B, and C) were also compared with that of the hematin standard to illustrate the effect of associated peptides on the solubility of heme iron at various pH values.

As previously illustrated in Figure 2, the solubility profile of HIP with DH between 8 and 15% at a 0.5 h digestion appeared to be similar between batches. However, the solubility at pH 2 was the major concern because this is the gastric condition where heme polymerization and precipitation occur. This subsequently limits the bioavailability of heme. In order to investigate whether higher DH would hamper HIP solubility, especially at pH 2, the solubility profile of HIP with low and high DH from the same batch were assessed at various pHs. 

**Figure 3:**
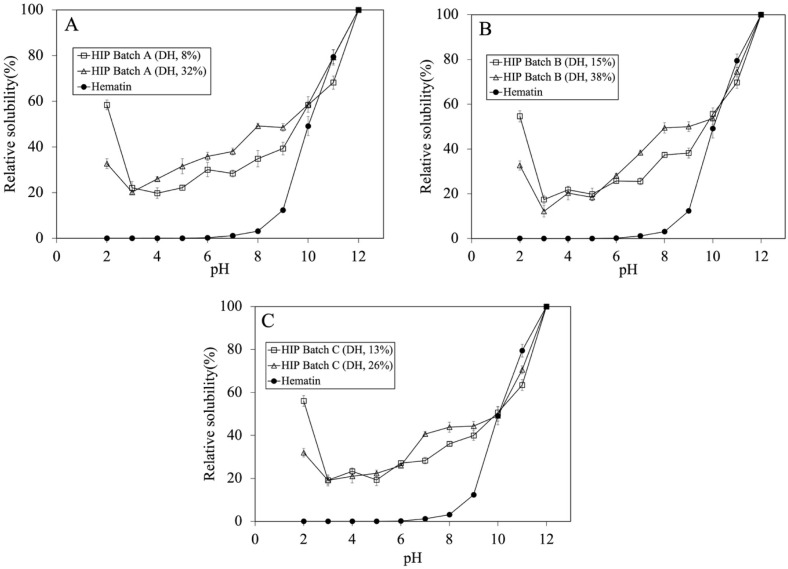
Tuna-HIP at different DH (not greater than 15% and not greater than 38%) from three different batches and hematin standard solubility at pH 2–12. (**A**–**C**) represent each tuna-HIP from Batch (**A**–**C**), respectively. Statistical analysis was performed using *t*-test, *p* = 0.05, comparing within the same batches.

The solubility profiles of HIP with low and high DH were investigated and are illustrated in Figure 3A–C. For batch A, the HIP with low DH showed a 1.57 times higher solubility at pH 2 than that with a 32% DH. However, the effect of low and high DH did not show significant solubility within the same batch at the pH between 3 and 6. A similar solubility profile trend was seen for Batches B and C. The most significant difference in the solubility at lower DH was at pH 2.

The trend of our tuna-HIP solubility at pH 2 to 12 was quite different when compared to that of fish-HIP [5]. The fish-HIP from yellowtail fish (*Seriola quinqueradiata*) showed the result of high solubility around 80% at pH 2–3, below 20% at pH 4–6, and above 80% at pH 7–12. The different findings between our tuna-HIP and fish-HIP [5] might be explained by many possible reasons. In the yellowtail fish-HIP study, fresh blood and concentrated red blood cells to produce HIP were utilized, and also an enrichment process was employed to isolate only hydrophobic peptides that can form a complex with heme iron through hydrophobic interactions, thereby increasing heme solubility especially at pH 2 and pH 7. In contrast, our tuna-HIP contained not only a hemoglobin-derived peptide, but also other peptides from other proteins in the blood (both hydrophilic and hydrophobic types), and no enrichment technique was applied. These aspects could lead to the difference in solubility properties where our HIP (DH between 8 and 15%) exhibited a 60% solubility at pH 2 and about 50% at pH 7.

Regarding HIP from bovine, the lowest solubility of bovine HIP was found at pH 5.0, and it was suggested that this pH is an average isoelectric point of HIP mixtures [6,10]. However, in our study, the tuna-HIP peptides might not have come from only hemoglobin but contain various types of proteins from the plasma. This might have led to different isoelectric points in tuna HIP. 

**Figure 4:**
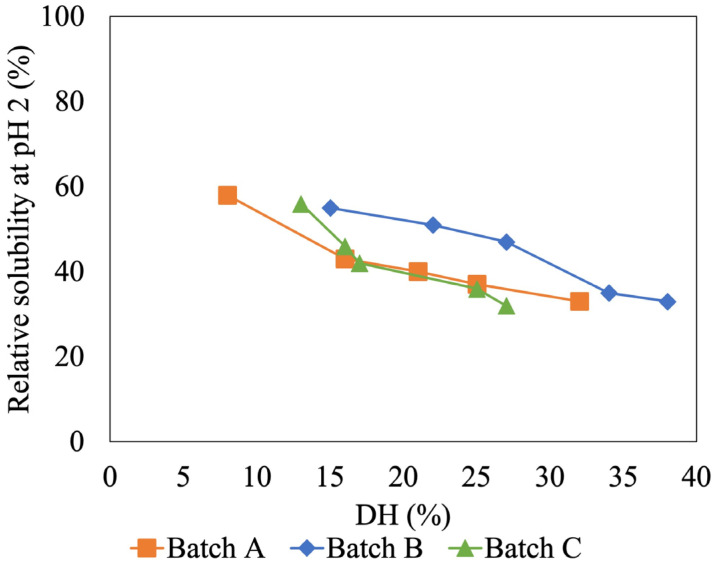
Effect of degree of hydrolysis on HIP solubility of (Batch A, B, and C) at pH 2.

Since the low and high %DH showed great different solubility at pH 2, the solubility of HIP with different %DH values was further investigated at this pH. This is a crucial pH for heme iron absorption as free heme molecules are insoluble at pH 2 (i.e., gastric pH), thus having a negative impact on absorption [9]. Figure 4 shows the relation between %DH and solubility of tuna-HIP at pH 2. The result showed that when the %DH of tuna-HIP increased, the relative solubility of tuna-HIP decreased. The highest solubility (around 60%) was obtained from our tuna-HIP when the DH was less than 15% (Figure 4). The trend of the relation between %DH and solubility of tuna-HIP in three batches confirmed that the solubility of low DH was not affected by batch variation.

Our findings are consistent with those of other in vitro digestion studies [7,9]. These studies revealed that the highest heme solubility at pH 2 was achieved at hydrolysis degrees ranging from 8 to 12% and the size of peptides ranging from 1 to 7.5 kDa. However, small peptides could not prevent heme polymerization [9]. Nevertheless, this study might provide target %DH values within the range of 7–15% for the production of HIP from tuna blood via enzymatic hydrolysis. 

**Figure 5:**
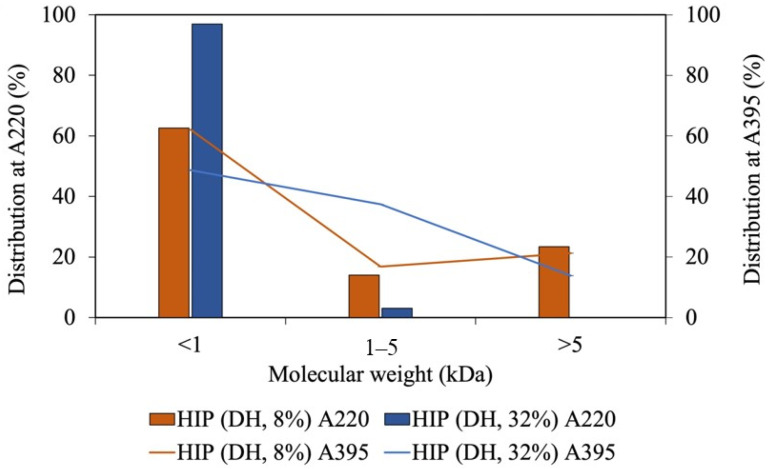
The molecular weight of tuna-HIP from low-DH (DH, 8%) and high-DH (DH, 32%) at A220, which monitors peptides, and A395, which monitors heme.

Since our tuna-HIP with an 8% DH from Batch A has the highest solubility at pH 2, it was selected to assess the molecular weight and the interaction between peptide and heme through size exclusion chromatography. The molecular species in HIP were analyzed by UV. The peptides were monitored at a UV of 220 nm and heme was detected at 395 nm. The molecular weight distributions of heme and peptides of the sample with DH of 8% and 32% are illustrated in Figure 5. The percent distribution was calculated by comparing area under the chromatogram of specific sizes to total area as illustrated in Supplementary Data. In both 8% and 32% DH, the majority of the peptides had a molecular weight of less than 1 kDa. At a 32% DH, the amount of peptides with less than 1 kDa was slightly higher than that of those produced by an 8% DH.

The heme–peptide complex was mainly found in the fraction with molecular weight of less than 1 kDa, followed by the size between 1 and 5 kDa. At higher DH, the populations with less than 1 kDa became larger, while those within the 1–5 kDa range became smaller. Since the fraction within the 1–5 kDa range was higher in an 8% DH, this group could contribute to the solubility enhancement at pH 2. A shorter peptide length displays a weaker interaction in the heme–peptide complex, which causes the impossibility to prevent the formation of large insoluble heme polymers [9]. Additionally, the decrease in the solubility of heme may be caused by the size of peptides and exposure of hydrophobic porphyrin rings with increased DH levels [10]. Heme can be separately detected from the peptide by absorption at 395 nm (Figure 5). The size-exclusion HPLC demonstrated that heme was also detected in the range of 1 to 5 kDa and at less than 1 kDa. Therefore, the size of peptides that interact with heme could be up to 50 amino acids with the molecular weight between 1 and 5 kDa. The high percentage distribution of heme observed in the 1 to 5 kDa fraction of the high-DH sample may be attributed to heme aggregation or dimerization, resulting in larger sizes (greater than 1.2 kDa) due to the small size of peptides in high DH. These peptides may be unable to prevent heme from aggregating through hydrophobic interactions.

**Figure 6:**
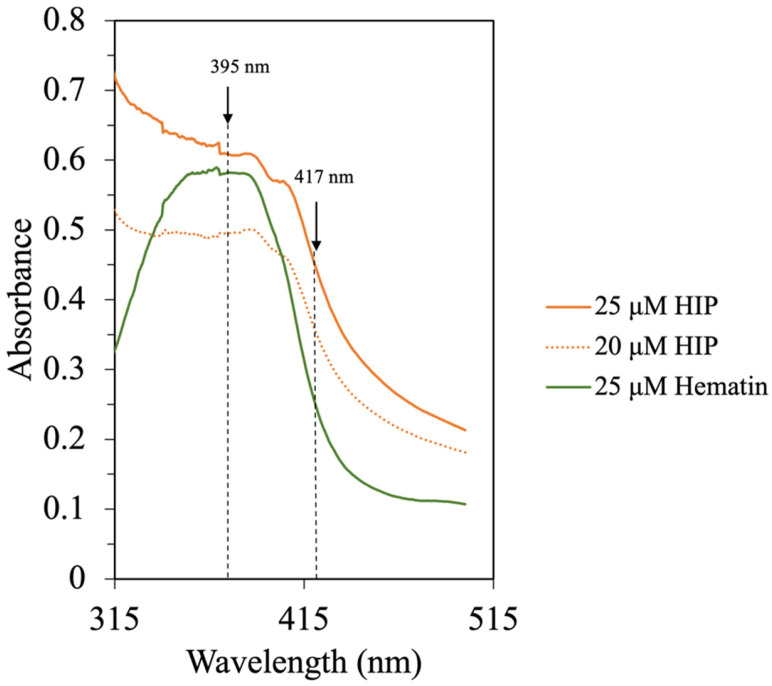
UV–Vis spectra of hematin and HIP in 0.01 M phosphate buffer, pH 7.5.

Lebrun, Bazus [6] isolated an 11% DH heme–peptide fraction from a bovine blood hydrolysate with enhanced heme solubility at acidic pH. In the mixture, peptides with approximately 3 kDa (30 amino acids)-long chains were identified to be associated with heme [6]. This is the same range (1–5 kDa) primarily found in this study at an 8% DH. In addition, fish-HIP, bovine-HIP, and porcine-HIP, which have effective solubility at acidic pH, were demonstrated to have molecular weight ranges of HIP of around 2–3.6 kDa, 4–5 kDa, and 1–7.5 kDa, respectively [5,6,7]. It should be noted that the molecular weight of heme is 634.5 g/mol, which could co-elute with short peptides having similar molecular weight less. In order to confirm the presence of the heme–peptide complex, the absorption spectra in the UV–visible region were conducted. Since the molecular weight of HIP from various species was reported to be different, the heme–peptide interaction was further confirmed by UV spectra as illustrated in Figure 6. 

Purified heme has a broad absorption spectrum between 315 nm and 400 nm with the center around 363 nm. This is also the indicator of heme polymerization [6]. The presence of peptides in the HIP fraction resulted in a shift of the Soret band with an increase in the absorption at 417 nm. In this study, the UV–Vis spectra of hematin and HIP at different concentrations in a 0.01 M phosphate buffer, pH 7.5, can be seen in Figure 6. The UV–Vis spectrum of hematin at pH 7.5 exhibited a broad Soret band centered at 370 nm. On the other hand, the UV–Vis spectrum of HIP showed a broad Soret band at 390 nm and a shift to 417 nm (Figure 6). These results were in good agreement with the bovine HIP study of Lebrun, Bazus [6]. The authors found that the Soret band of hemin at pH 7.5 was centered at 363 nm, suggesting that this band comes from intermolecular heme–heme association due to porphyrins being known to polymerize in an aqueous solution. Moreover, they also found that the Soret band of their hemin–peptide complex shifted to 417 nm due to heme–peptide interaction and the dissociation of heme to a monomer [6]. These results indicated that our tuna-HIP could be in a heme–peptide complex form and supported the observation that tuna-HIP has high solubility at pH 2.

The degree of hydrolysis increased with time as a result of Alcalase hydrolysis activity. Different %DH represent different peptides in various sizes and can affect HIP solubility. The solubility pattern between HIP from different DHs showed that with increasing the DH, the solubility at pH 2 decreased because of an increase in the number of small peptides and the amount of exposed hydrophobic porphyrin rings in HIP at high DH [6,10]. In simulated stomach conditions (pH 2), tuna-HIP hydrolyzed for 0.5 h (DH = 8–15%) demonstrated the highest solubility, which is related to iron absorption. This finding might be useful for the commercial production of high-solubility HIP for iron supplementation in the future due to the short hydrolysis time when compared to previous studies (Table 4).

## 4. Conclusions

Tuna blood could be used as a starting raw material for the preparation of tuna-HIP. Since the tuna is processed in mass quantities and also from a frozen condition, the batch variations might influence the final HIP key compositions including protein, total iron, heme, and heme iron. This study showed that although different batches might lead to different protein contents, batch variation does not affect other tuna-HIP characteristics (i.e., total iron, heme, and heme iron) and solubility. The tuna-HIP solubility at the gastric pH 2 appears to depend on the peptide molecular weight, which resulted from different degrees of hydrolysis. Here, the tuna-HIP with the highest solubility at pH 2 was achieved at 0.5 h, resulting in tuna-HIP with a DH of about 8–15% and molecular weights at <1 kDa and 1–5 kDa. At this digestion time, batch variation has no effect on the DH and the pH solubility profiles (pH 2–12), making it a potential parameter for quality control. However, due to the freeze–thawing processing of tuna, tuna blood cannot be processed in the same way as fresh blood to generate HIP. Without pre-concentration of red blood cells, the peptides present in tuna-HIP could include various mixed proteins present in the tuna blood and lead to reduced iron concentrations in tuna-HIP when compared with other fresh blood sources. Nevertheless, tuna-HIP still has potential as the solubility is on par with (or better than) other blood sources, and much better than heme molecules alone.

Lastly, in order to develop tuna-HIP into a food supplement, further studies in various aspects are required. These include biological properties of tuna-HIP including bioavailability, absorption, and clinical studies in normal and anemic populations. Regarding the industrial aspect, pilot-scale production and factors associated with quality control and assurance must be investigated. These included batch-to-batch consistency, shelf life under normal and accelerated condition, formulation, process design and development.

## Figures and Tables

**Table 1:** Three different batches of skipjack tuna used in this study.

Tuna Batch	Fishing Trip Date	Ocean Area	Number of Tuna Fish
Batch A	Jan.–Feb. 2022	Western Central Pacific (FAO Major Fishing Area 71)	40
Batch B	Feb.–Mar. 2022	Western Central Pacific (FAO Major Fishing Area 71)	40
Batch C	Jan.–Feb. 2022	Western Central Pacific (FAO Major Fishing Area 71)	40

Note: Batch A and Batch C were from different catch vessels fishing in the same time period. FAO: Food and Agriculture Organization of the United Nations.

**Table 2:** Composition of tuna blood from three different batches.

Composition (Dry Basis)	Batch A	Batch B	Batch C
Protein (g/100 g)	58.78 ± 0.56 ^a^	63.51 ± 1.65 ^b^	65.99 ± 0.41 ^c^
Iron (mg/100 g) ^ns^	70.54 ± 3.22	70.27 ± 1.79	69.97 ± 2.21
Total iron (%) ^ns^	0.07%	0.07%	0.07%
Heme iron ** (mg/100 g) ^ns^	54.19 ± 0.38	54.76 ± 0.24	52.60 ± 0.55
Total Heme iron (%) ^ns^	75.88 ± 3.24	77.99 ± 3.15	74.57 ± 3.66

Values are given as means ± SD of triplicate blood sampling. ** Heme iron was calculated from 1 μg of hematin containing 0.0882 μg iron. ^ns^ Means no significant difference among three batches (One-way ANOVA test, *p* > 0.05). a, b, and c mean significant difference among three batches, *p* < 0.05.

**Table 3:** Effect of tuna batch variation and hydrolysis on iron content and stability.

Composition (Dry Basis)	Batch A		Batch B		Batch C	
	Blood	HIP (4 h)	Blood	HIP (4 h)	Blood	HIP (4 h)
Iron (mg/100 g) ^ns^	70.54 ± 3.22	67.93 ± 0.60	70.27 ± 1.79	70.92 ± 0.61	69.97 ± 2.21	67.71 ± 2.66
Heme iron (mg/100 g) ^ns^	54.19 ± 0.38	54.25 ± 0.61	54.76 ± 0.24	55.06 ± 0.38	52.60 ± 0.55	51.71 ± 0.59

Values are given as means ± SD of triplicate HIP powder sampling. Heme iron was calculated from 1 μg of hematin containing 0.0882 μg iron. ^ns^ means no significant difference (Paired sample *t*-test, *p* > 0.05).

**Table 4:** HIP molecular weight and solubility of different sources of blood.

Sources	Enzyme Conditions	%DH	Molecular Weight	Solubility	References
Skipjack tuna	Alcalase, pH 8.5, 0.5 h	8–15%	<1 kDa and 1–5 kDa	pH 2, pH > 7	This study
Bovine	Pepsin, pH 4, 24 h	11%	4–5 kDa	pH 2–12 except 5.5	[6]
	Pepsin, Subtilisin	15%	N/A	pH2	[9]
	Esparase + Flavourzyme	19.8%	N/A	pH > 6	[10,32]
Porcine	Alcalase	N/A	80 kDa and 250 kDa	Wide range pH (N/A specific pH)	[33]
	Alcalase + Flavourzyme, pH 7.5	8–12%	1–7.5 kDa	pH 2	[7]
	Neutrase, acid enzyme, alkaline enzyme, and Papain	6–12%	3–14.4 kDa	pH > 8	[8]
Yellowtail fish	Alcalase, pH 10, 24 h	N/A	2–3.6 kDa	pH < 4, pH > 6	[5]

N/A: not available (data were not mentioned by the authors).

## Data Availability

Data are contained within the article.

## Supplementary Materials

The following supporting information can be downloaded at: https://www.mdpi.com/article/10.3390/foods12173249/s1, Figure S1: Thyroglobulin (669 kDa) chromatogram at 220 nm, Figure S2: Cytochrome C (12 kDa) from bovine heart chromatogram at 220 nm, Figure S3: Aprotinin from bovine lung (MW: 6511 Da) chromatogram at 220 nm, Figure S4: Bacitracin (1423 Da) chromatogram at 220 nm, Figure S5: N-Hippuryl-His-Leu (MW: 429 Da) chromatogram at 220 nm, and Figure S6: Gly-Gly (MW: 132 Da) standard chromatogram at 220 nm.
