# Supplementary material for: Production and Characterization of Heme Iron Polypeptide from the Blood of Skipjack Tuna (Katsuwonus pelamis) Using Enzymatic Hydrolysis for Food Supplement Application
Source: Foods. 2023 Aug 29;12(17):3249. doi: 10.3390/foods12173249 (PMC10486430; doi:10.3390/foods12173249)
Supplement: Supplementary file 1 [file foods-12-03249-s001.zip › foods-2568216-supplementary.pdf]

Supplementary data

Protein Standard Chromatogram

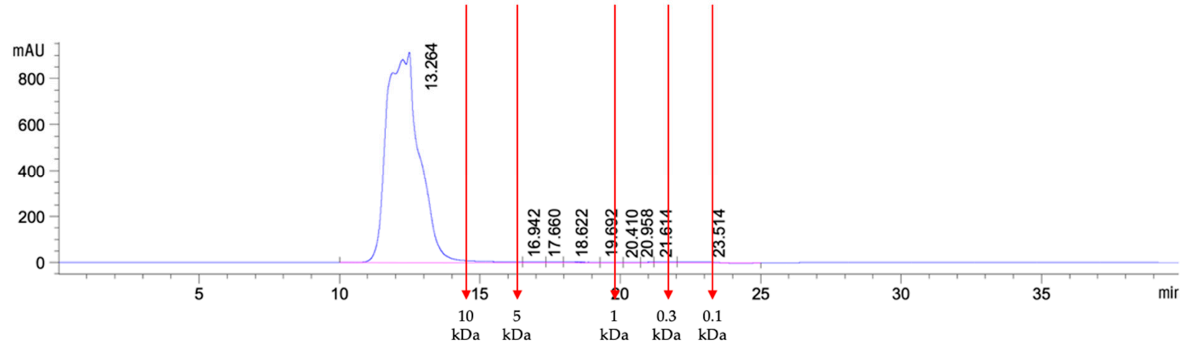

Figure S1. Thyroglobulin (669 kDa) chromatogram at 220 nm.

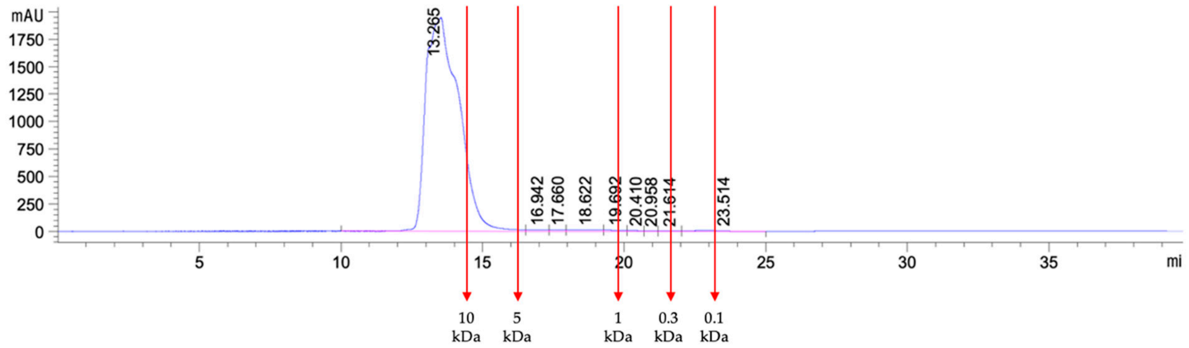

Figure S2. Cytochrome C (12 kDa) from bovine heart chromatogram at 220 nm.

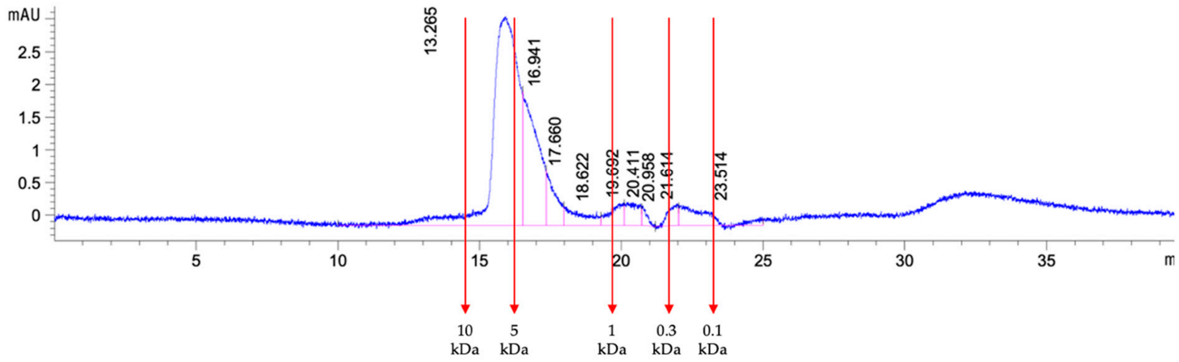

Figure S3. Aprotinin from bovine lung (MW: 6511Da) chromatogram at 220 nm.

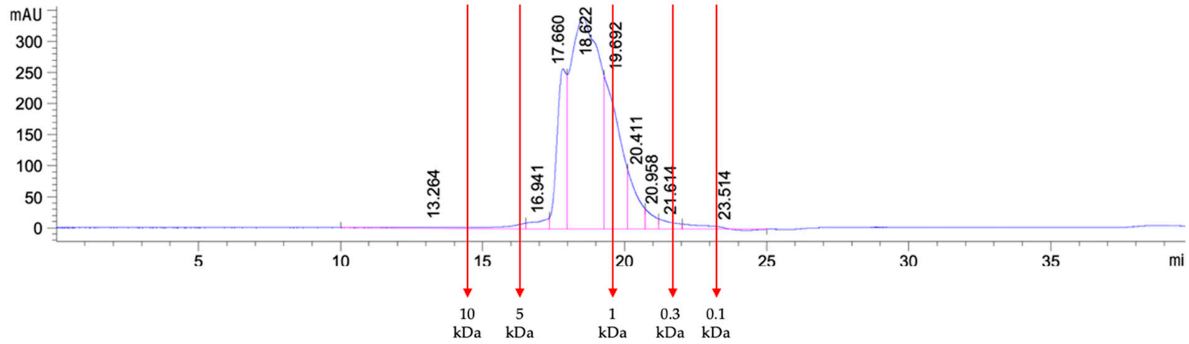

Figure S4. Bacitracin (1423 Da) chromatogram at 220 nm.

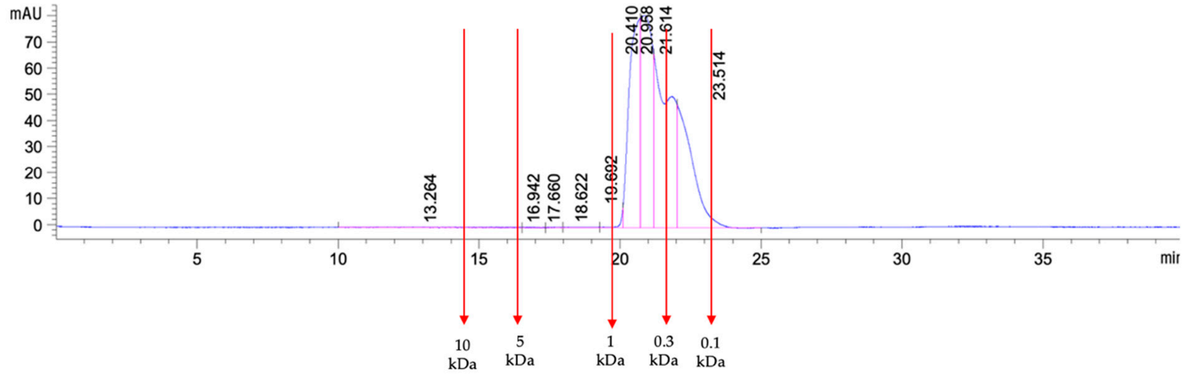

Figure S5. N-Hippuryl-His-Leu (MW: 429 Da) chromatogram at 220 nm.

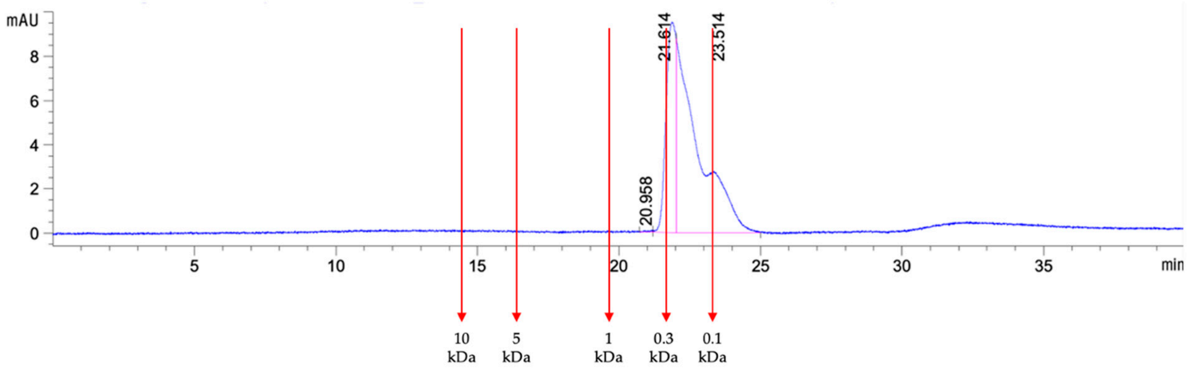

Figure S6. Gly-Gly (MW: 132 Da) standard chromatogram at 220 nm.
